# Trastuzumab Sensitizes Ovarian Cancer Cells to EGFR-targeted Therapeutics

**DOI:** 10.1186/1757-2215-3-7

**Published:** 2010-03-27

**Authors:** Jason A Wilken, Kristy T Webster, Nita J Maihle

**Affiliations:** 1 Yale University, School of Medicine, Department of Obstetrics, Gynecology, and Reproductive Sciences, PO Box 208063, 310 Cedar Street, New Haven, CT 06520-8063, USA; 2 Yale University, Department of Molecular, Cellular, and Developmental Biology, New Haven, CT 06520-8063, USA; 3 University of Massachusetts, School of Medicine, 55 Lake Avenue North, Worcester, MA, 01605, USA; 4 Yale University, School of Medicine, Departments of Pathology and Pharmacology, PO Box 208063, 310 Cedar Street, New Haven, CT 06520-8063, USA

## Abstract

**Background:**

Early studies have demonstrated comparable levels of HER2/ErbB2 expression in both breast and ovarian cancer. Trastuzumab (Herceptin), a therapeutic monoclonal antibody directed against HER2, is FDA-approved for the treatment of both early and late stage breast cancer. However, clinical studies of trastuzumab in epithelial ovarian cancer (EOC) patients have not met the same level of success. Surprisingly, however, no reports have examined either the basis for primary trastuzumab resistance in ovarian cancer or potential ways of salvaging trastuzumab as a potential ovarian cancer therapeutic.

**Methods:**

An in vitro model of primary trastuzumab-resistant ovarian cancer was created by long-term culture of HER2-positive ovarian carcinoma-derived cell lines with trastuzumab. Trastuzumab treated vs. untreated parental cells were compared for HER receptor expression, trastuzumab sensitivity, and sensitivity to other HER-targeted therapeutics.

**Results:**

In contrast to widely held assumptions, here we show that ovarian cancer cells that are not growth inhibited by trastuzumab are still responsive to trastuzumab. Specifically, we show that responsiveness to alternative HER-targeted inhibitors, such as gefitinib and cetuximab, is dramatically potentiated by long-term trastuzumab treatment of ovarian cancer cells. HER2-positive ovarian carcinoma-derived cells are, therefore, not "unresponsive" to trastuzumab as previously assumed, even when they not growth inhibited by this drug.

**Conclusions:**

Given the recent success of EGFR-targeted therapeutics for the treatment of other solid tumors, and the well-established safety profile of trastuzumab, results presented here provide a rationale for re-evaluation of trastuzumab as an experimental ovarian cancer therapeutic, either in concert with, or perhaps as a "primer" for EGFR-targeted therapeutics.

## Background

Cancer diagnostics and treatment are being revolutionized by the clinical application of information generated during the past three decades of basic cancer research. Some of the most difficult to treat malignancies have been shown to have predictable alterations in key signal transduction pathways, and the discovery of these abnormalities has allowed the development of improved, side-effect sparing biologically-targeted therapeutics. Examples of these novel drugs include imatinib (Gleevec), trastuzumab (Herceptin), gefitinib (Iressa) and erlotinib (Tarceva), cetuximab (Erbitux) and panitumumab (Vectibix), and sunitinib (Sutent), which have been FDA approved for the treatment of chronic myelogenous leukemia, HER2-positive breast cancer, non-small cell lung cancer, colorectal cancer, and gastrointestinal stromal and advanced kidney cancer, respectively. Each of these drugs targets the specific kinase machinery on which tumor cell growth is dependent. Despite the impressive responsiveness of certain types of cancers to these new drugs, resistance to many of these new drugs remains a serious clinical obstacle. Nowhere is this more evident than in advanced epithelial ovarian cancer (EOC), the leading cause of death in women with gynecological malignancies in the United States [1], for which only incremental improvements in chemotherapy have been achieved over the past several decades [2].

No biologically-targeted drugs have been approved for the treatment of EOC. This is despite the observation that many candidate signaling proteins, including receptor tyrosine kinases of the EGFR/ErbB/HER family, are frequently expressed in these tumors. The EGFR/ErbB/HER family of receptor tyrosine kinases (i.e., ErbB1/HER1/EGFR, ErbB2/HER2/neu, ErbB3/HER3, ErbB4/HER4) has been documented to play fundamental roles in normal ovarian development, follicle maturation, ovulation, and tissue homeostasis [3]. It is, therefore, not surprising that overexpression of HER family members is common in ovarian tumors and ovarian carcinoma-derived cell lines. Yet, recent clinical trials targeting EGFR with cetuximab [4-6], matuzumab [7], gefitinib [8], and erlotinib [9] in EOC patients have shown only modest clinical responsiveness http://www.gog.org.

Perhaps most surprising is the failure of HER2-targeted therapeutics in the treatment of ovarian cancer patients. Trastuzumab (Herceptin) is a therapeutic antibody that targets HER2; it is a well-tolerated drug [10] and has proven exceptionally useful in the treatment of HER2-positive breast cancer [11]. A small number of early clinical trials suggested that trastuzumab would not be an effective treatment option for EOC patients [12,13], despite the negative correlation between HER2 expression and survival in EOC patients [14]. Consequently, trastuzumab use, even for further clinical study, has quickly lost favor as an experimental therapeutic for the treatment of ovarian cancer patients.

We and others previously have demonstrated that HER receptor tumor cell expression, as currently measured, is not an accurate positive predictor of responsiveness to HER-targeted therapeutics [5,9,15]. Here we further demonstrate that growth inhibition of ovarian cancer cells is not an accurate metric of HER-targeted drug "responsiveness." Specifically, we demonstrate that long-term trastuzumab treatment of HER2-positive EOC-derived cells confers de novo sensitivity to EGFR-targeted therapeutics, regardless of trastuzumab's ability to inhibit cell growth. We propose these results warrant re-evaluation of the very definition of "trastuzumab resistance." Moreover, since so-called 'resistant' EOC cells are, in fact, primed by trastuzumab to acquire de novo sensitivity to other HER-targeted therapeutics, we propose that these results provide the rationale for re-evaluation of trastuzumab as an experimental ovarian cancer therapeutic, perhaps as a priming agent for EGFR-targeted drugs.

## Methods

### Reagents and cell lines

Ovarian carcinoma cell lines A1847, A2780 (and cisplatin-resistant subclones A2780 CP30 and A2780 CP70), BG-1, ES-2, MDAH-2774, OVCAR-7, OVCAR-10, PEO-1, PEO-4, and UPN-251 were a obtained from Dr. D. Connolly, OVCA-429, OVCA-432, and OVCA-433 were obtained from Dr. R. Bast, Jr., IGROV-1 and OVCAR-8 were obtained from Dr. W. Cliby, SKOV-6 and SKOV-8 were a obtained from Dr. C. Marth, and the HEY cell line was obtained from Dr. R. Buick. OVCAR-3, and the breast carcinoma cell lines BT-474 and SKBR-3 were purchased from the American Tissue Culture Collection. Chinese hamster ovary (CHO) cells stably expressing exogenous HER2 under the CMV promoter (CHO-HER2) were established by Drs. H. J. Lee and Maihle (unpublished result). Anti-EGFR (sc-03), anti-HER3 (sc-285), and anti-HER4 (sc-283) antibodies were purchased from Santa Cruz Biotechnologies. Anti-HER2 (Ab-1) antibody was purchased from NeoMarkers, Inc. Function-blocking anti-HER3 antibody (H3.105) was purchased from Upstate Biologicals. Anti-β-tubulin antibody was purchased from Cell Signaling Technology. Cell culture media and all culture supplements were purchased from Mediatech, except for fetal bovine serum (FBS), which was purchased from Atlanta Biologicals, and G418, which was purchased from GibcoBRL. Cetuximab was obtained from Bristol Myers Squibb, trastuzumab was obtained from Genentech, and erlotinib, gefitinib, and lapatinib were obtained from Chemitek. Bovine serum albumin, fraction V (BSA) and human transferrin were purchased from Sigma-Aldrich. A colormetric WST-1-based cell proliferation assay was purchased from Roche Diagnostics.

### Cell culture

All media formulations were supplemented with 10% FBS, 100 U/ml penicillin, 100 μg/ml streptomycin, and 2 mM L-glutamine. A1847, A2780, OVCAR-3, OVCAR-7, OVCAR-10, PEO-1, PEO4, and UPN-251 were cultured with RPMI 1640. BG-1 and HEY cells were cultured with DMEM/Ham's F12. CAOV-3, IGROV-1, MDAH-2774, OVCAR-5, OVCAR-8, and SKBR-3 cells were cultured with DMEM. ES-2 and SKOV-3 cells were cultured with McCoy's 5A. BT-474, OVCA-429, OVCA-432, OVCA-433, SKOV-3, and SKOV-6 cells were cultured with Eagle's MEM supplemented with 1 mM sodium pyruvate and non-essential amino acids. CHO-HER2 were cultured with Ham's F12, supplemented with 800 μg/ml G418.

### Immunoblot analysis of HER expression

Confluent or near-confluent dishes of cells were rinsed with phosphate buffer (PBS; 4°C) and harvested by cell scraping, followed by resuspension with PBS (4°C) and brief centrifugation. Cell pellets were lysed by boiling with 2.5% SDS, 0.5% sodium deoxycholate, and 0.5% NP-40 for 10 minutes. Protein concentrations in cell lysates were determined using the Bio-Rad DC assay. Cell lysates, normalized by protein content, were resolved by 7.5% polyacrylamide gel electrophoresis in the presence of 0.1% SDS. Gel proteins were transferred to polyvinyl difluoride membrane by semi-dry immunoblot (Millipore), followed by blocking with TBS (10 mM Tris HCl, 150 mM NaCl, pH 7.4) prepared with 5% non-fat dry milk for one hour at room temperature. Membranes were rinsed six times for five minutes each with TBS with 0.1% Tween 20 (TBS-TW20), and incubated with TBS with 1% BSA and primary anti-EGFR (1:500 dilution), anti-HER2 (1:4000 dilution), anti-HER3 (1:250 dilution), or anti-HER4 (1:500 dilution) overnight at 4°C. Membranes were rinsed six times for ten minutes each with TBS-TW20 and incubated with goat anti-rabbit horseradish peroxidase conjugated secondary antibody (Pierce, 1:4000 dilution) for one hour at room temperature. Membranes were rinsed six times for ten minutes each, and chemiluminescnce was visualized with a NucleoVISION camera station following incubation with enhanced chemiluminescent (ECL) reagent (Pierce).

### Long-term trastuzumab treatment of ovarian cell lines

A1847, IGROV-1, OVCAR-7, and SKOV-3 cells were cultured with (T100) or without (parental) 100 μg/ml trastuzumab for 12 weeks in RPMI 1610 media, supplemented with 10% FBS, 100 U/ml penicillin, 100 μg/ml streptomycin, 2 mM L-glutamine, and 1 mM sodium pyruvate. Confluent or near-confluent flasks of cells were passaged by treatment with 0.25% trypsin, and cells were resuspended and transferred to a new flask at a 1:10 dilution.

### Effect of HER inhibitors on ovarian cell line growth

Parental and T100 A1847, IGROV-1, OVCAR-7, and SKOV-3 cells were seeded into 96 well plates at a concentration of 2.5 × 10^3 ^cells/50 μl of assay medium consisting of RMPI 1610 media supplemented with penicillin/streptomycin, L-glutamine, sodium pyruvate, 0.02% BSA, and 10 μg/ml human transferrin (assay media). After overnight incubation in serum free media, 50 μl of assay media supplemented with 10% FBS, and either 2 μM gefitinib, 2 μM erlotinib, 2 μM lapatinib, 400 μg/ml cetuximab, or 20 μg/ml H3.105 was added to each well in quintuplicate. Cell proliferation was measured after 120 hours using a colormetric WST-1-based assay (n = 1).

## Results

### HER2-expression in EOC-derived cell lines is not correlated with trastuzumab mediated growth inhibition

**Figure 1:**
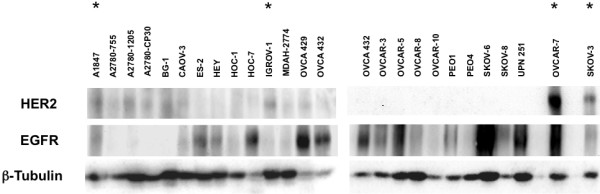
**Selection of HER2 positive ovarian carcinoma-derived cell lines**. Cell lysates, normalized for protein content, were probed following SDS-PAGE with anti-HER2 and anti-EGFR antibody. A1847 and IGROV-1 cells, which express moderate levels of HER2, and OVCAR-7 and SKOV-3, which express high levels of HER2, were selected for further study. Cell lines selected for further study are noted with an asterisk. Tubulin expression is included as a loading control. These blots are representative of two successive passages of ovarian carcinoma-derived cell lines.

HER2 expression was assayed in a large panel of EOC-derived cell lines. As shown in Figure 1, the cell lines SKOV-3 and OVCAR-7 expressed the highest levels of HER2, whereas A1847 and IGROV-1 expressed moderate levels of HER2. IGROV-1 and SKOV-3 both have been reported previously to express moderate to high levels of HER2, respectively [3], while HER2 expression in A1847 and OVCAR-7 has not been reported previously.

**Figure 2:**
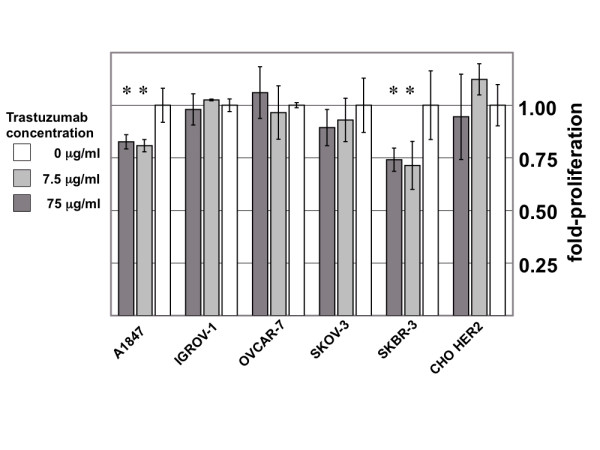
**Trastuzumab sensitivity does not correlate with HER2 expression**. A1847, IGROV-1, OVCAR-7, SKOV-3, SKBR-3, and CHO HER2 cells were exposed to trastuzumab (0-75 μg/mL) for 120 hours, and cell proliferation was measured by a WST-1-based colormetric assay. A1847 and SKBR-3 cells were significantly growth inhibited by trastuzumab while IGROV-1, OVCAR-7, SKOV-3, and CHO HER2 cells were not significantly growth inhibited by trastuzumab. Student's T-test was used to determine whether significant differences in cell proliferation exist between untreated and treated cell populations. Asterisk denotes statistical variances (p < 0.05) where treated cells were growth inhibited.

To determine whether HER2 expression might be correlated with trastuzumab sensitivity, the A1847, IGROV-1, OVCAR-7, and SKOV-3 cell lines were treated with increasing doses of trastuzumab in a cell proliferation assay. As shown in Figure 2, A1847 was modestly growth inhibited by trastuzumab, whereas IGROV-1, OVCAR-7, and SKOV-3 were not growth inhibited, despite the wide range of HER2 expression levels among this subset of cell lines. In agreement with previous reports [16,17], SKBR-3, a HER2-overexpressing breast-cancer cell line, included here as a positive control, was growth inhibited by trastuzumab (Fig. 2). In addition, the well-studied HER2-positive breast cancer cell line BT-474 was >50% growth inhibited by 10 μg/ml trastuzumab (data not shown). Notably, CHO cells stably expressing exogenous HER2 (CHO-HER2), but which express no other endogenous HER family member, also were not growth inhibited by trastuzumab (Fig. 2). We, therefore, conclude that trastuzumab-mediated growth inhibition is not strictly correlated with HER2 expression in the ovarian carcinoma-derived cell lines studied in this panel. This counter-intuitive observation prompted us to evaluate whether long-term trastuzumab treatment might have other measurable effects relevant to the expression and/or function of related HER family members in these cell lines, as described in greater detail below.

### Long-term trastuzumab treatment induces moderate changes in HER expression

**Figure 3:**
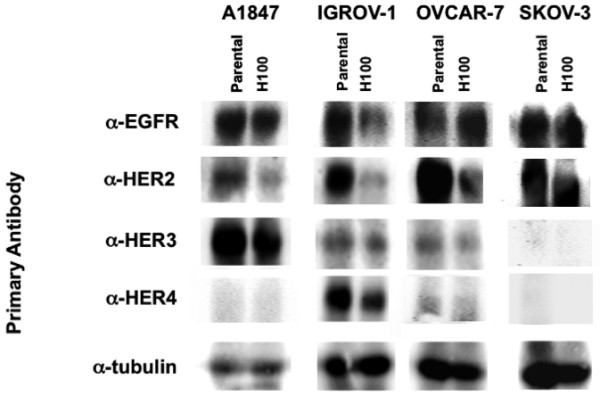
**HER expression reprogramming in ovarian carcinoma-derived cell lines following long-term trastuzumab treatment**. Immunoblot of epidermal growth factor receptor (EGFR), HER2, HER3, and HER4 expression in parental vs. T100 ovarian carcinoma cell lines A1847, IGROV-1, OVCAR-7, and SKOV-3. Lysates of parental and T100 subclones, normalized for protein content, were probed following SDS-PAGE with α-EGFR, -HER2 -HER3, -HER4, and -tubulin. Note, enhanced chemiluminescent exposures were calibrated for each cell line to allow for accurate assessment of individual HER receptors; the HER expression patterns presented here are therefore not comparable between lanes.

In an effort to model long-term trastuzumab treatment of ovarian cancer in vitro, all four HER2-positive ovarian cancer cell lines, i.e., A1847, IGROV-1, OVCAR-7, and SKOV-3 were cultured continuously for 12 weeks in the presence (T100) or absence (parental) of 100 μg/ml trastuzumab, well within the range of serum trastuzumab concentrations observed in EOC patients treated with trastuzumab in a phase II clinical trial [12]. Lower trastuzumab concentrations were used for sensitive cell lines, reaching 100 μg/ml by week six. Expression of all four HER receptor family members was assessed in parental vs. T100 cells by immunoblot analysis. In agreement with previous reports, A1847 expressed moderate levels of EGFR [18], IGROV-1 expressed moderate levels of both EGFR and HER-2 [19], SKOV-3 expressed moderate levels of EGFR, high HER-2, and low HER-3 and HER-4 [20]. Expression of HER-2, HER-3, and HER-4 in A1847, HER-3 and HER-4 in IGROV-1, or any HER-family member in OVCAR-7 has not been reported previously. Figure 3 illustrates the modest alteration of HER receptor expression in some T100 cells compared to parental cells; similar changes in the pattern of HER expression have been reported in HER2-positive breast and mouse fibroblast derived cell lines following treatment with trastuzumab [15,21].

### Trastuzumab induces responsiveness to EGFR-targeted therapeutics

The observation that HER expression levels are variously altered in T100 cells compared to parental cell lines led us to hypothesize that T100 cells might also differ in their growth-inhibitory response to HER-targeted inhibitors relative to parental controls. All four T100 cell lines and their corresponding parental counterparts were treated with 1 μM gefitinib, 1 μM erlotinib, 1 μM lapatinib, or 200 μg/ml cetuximab for 120 hours; these concentrations are at or below the steady-state peak serum concentrations observed in treated cancer patients [22-25].

As summarized in Table 1, each of these FDA-approved HER-targeted therapeutics effectively inhibited the growth of certain T100 cells. For example, erlotinib and lapatinib inhibited proliferation of all four tested cell lines, whereas gefitinib inhibited the proliferation of A1847 and SKOV-3 cells, and cetuximab inhibited the proliferation of OVCAR-7 and SKOV-3. Furthermore, subsets of T100 cells acquired de novo sensitivity to one or more of these FDA-approved drugs: IGROV-1 T100 cells and OVCAR-7 T100 cells acquired de novo sensitivity to gefitinib, and IGROV-1 T100 cells and A1847 T100 cells acquired de novo sensitivity to cetuximab (Table 1; values in bold).

**Table 1:** HER inhibitors and cell proliferation in parental vs. T100 ovarian cell lines.

	A1847		IGROV-1		OVCAR-7		SKOV-3	
	
	Parental	T100	Parental	T100	Parental	T100	Parental	T100
**Gefitinib**								
0 μM	1.000 ± 0.055	1.000 ± 0.008	1.000 ± 0.017	1.000 ± 0.057	1.000 ± 0.010	1.000 ± 0.034	1.000 ± 0.008	1.000 ± 0.002
1 μM	0.812 ± 0.006	0.829 ± 0.040	**0.998 ± 0.039**	**0.691 ± 0.026**	**0.977 ± 0.125**	**0.899 ± 0.060**	0.797 ± 0.060	0.884 ± 0.022
	p = 0.001	p = 0.0005	p = 0.93	p = 0.0006	p = 0.96	p = 0.02	p = 0.001	p = 0.0002
**Erlotinib**								
0 μM	1.000 ± 0.075	1.000 ± 0.011	1.000 ± 0.041	1.000 ± 0.037	1.000 ± 0.072	1.000 ± 0.013	1.000 ± 0.027	1.000 ± 0.043
1 μM	0.741 ± 0.063	0.676 ± 0.053	0.753 ± 0.060	0.588 ± 0.046	0.612 ± 0.027	0.653 ± 0.096	0.625 ± 0.031	0.696 ± 0.023
	p = 0.0004	p < 10^-4^	p = 0.001	p < 10^-6^	p = 0.0007	p = 0.001	p = 10^-6^	p < 10^-27^
**Lapatinib**								
0 μM	1.000 ± 0.007	1.000 ± 0.027	1.000 ± 0.048	1.000 ± 0.062	1.000 ± 0.070	1.000 ± 0.023	1.000 ± 0.108	1.000 ± 0.047
1 μM	0.762 ± 0.063	0.645 ± 0.069	0.789 ± 0.098	0.666 ± 0.031	0.819 ± 0.084	0.673 ± 0.048	0.845 ± 0.023	0.889 ± 0.082
	p = 0.001	p = 0.0001	p = 0.005	p < 10^-4^	p = 0.006	p < 10^-5^	p = 0.03	p = 0.04
**Cetuximab**								
0 μg/ml	1.000 ± 0.091	1.000 ± 0.038	1.000 ± 0.075	1.000 ± 0.067	1.000 ± 0.090	1.000 ± 0.032	1.000 ± 0.031	1.000 ± 0.049
200 μg/ml	**0.974 ± 0.027**	**0.594 ± 0.110**	**0.892 ± 0.071**	**0.554 ± 0.067**	0.588 ± 0.050	0.657 ± 0.057	0.736 ± 0.038	0.854 ± 0.010
	p = 0.57	p = 0.0006	p = 0.07	p < 10^-5^	p < 10^-4^	p < 10^-4^	p < 10^-5^	p = 0.002
**H3.105**								
0 μg/ml	1.000 ± 0.071	1.000 ± 0.022	1.000 ± 0.050	1.000 ± 0.050	1.000 ± 0.011	1.000 ± 0.028	1.000 ± 0.040	1.000 ± 0.018
10 μg/ml	0.913 ± 0.095	0.989 ± 0.059	1.006 ± 0.132	0.979 ± 0.152	0.895 ± 0.030	0.856 ± 0.027	0.969 ± 0.039	1.048 ± 0.043
	p = 0.14	p = 0.71	p = 0.92	p = 0.78	p = 0.0006	p < 10^-4^	p = 0.28	p = 0.07

Parental vs. long-term trastuzumab treated (T100) A1847, IGROV-1, OVCAR-7, and SKOV-3 cells were treated with gefitinib, erlotinib, lapatinib, cetuximab, or H3.105 for 120 hours, and cell proliferation was measured by a WST-1-based colormetric assay. The inter-quartile rule was used to eliminate data outliers before calculating the mean absorbance for untreated and treated cell populations. Fold change in cell numbers is normalized against values determined for untreated cells. Student's T-test was used to calculate p values. Instances where T100 but not parental cell lines were significantly growth inhibited are highlighted in **bold**.

## Discussion

One assumption underlying the advent of 'personalized medicine' has been the concept of assessing the molecular characteristics of a patient's tumor in order to individually tailor a 'personalized' treatment strategy. Yet we and others clearly show that identification of a specific target molecule within a cell doesn't always correlate with successful cell growth inhibition by biologically-targeted therapeutics (e.g., CHO cells engineered to express HER2 are uneffected by trastuzumab treatment; Fig 2). Recent results across disease sites further suggest that it may be time to not only re-evaluate the accuracy of target gene expression assays, but also the potential importance of target gene expression itself in forecasting responsiveness to certain biologically-targeted therapeutics. The recent incongruity observed among EGFR-expressing colon cancer patients and responsiveness to cetuximab is a case in point. In these studies, K-Ras mutation status has proven to be a clinically useful *negative *indicator of responsiveness to cetuximab [26-29], but in no case is there a single accurate *positive *predictor of responsiveness to this new drug, including analysis of expression of cetuximab's target i.e., EGFR, using currently available methods. K-Ras, PTEN, c-Met, and mutations in the EGFR tyrosine kinase domain, but not overall EGFR expression, are associated with resistance to EGFR tyrosine kinase inhibitors erlotinib and gefitinib in lung cancer, as reviewed in [30,31]. More recently, Matulonis and colleagues demonstrated that tumor HER3 expression is a better predictor than HER2 for response to pertuzumab (a HER2-directed therapeutic antibody) in patients with platinum-resistant ovarian cancer [32]. Even in the well studied case of breast cancer, Paik et al., have shown that patients with tumors expressing even low levels of HER2 may gain benefit from trastuzumab therapy [33]. Together, these results are consistent with the notion that analysis of signaling networks and their aberrations may be better predictors of therapeutic response than is analysis of individual components within these networks.

In the case of EOC, for example, trastuzumab has not been shown to be effective in early clinical trials for the treatment of ovarian cancer patients. These disappointing results have been vexing since EOC tumors and EOC-derived cell lines express or overexpress HER family members at the same frequency as do many malignant breast tumors. Yet, if one examines the in vitro effects of trastuzumab, such results may be less surprising. For example, trastuzumab does not inhibit Akt activity in the ovarian carcinoma-derived cell line SKOV-3 [34] despite similar levels of HER2 expression as those observed in the breast carcinoma cell line SKBR-3 in which trastuzumab is a potent cell growth inhibitor [35]. Based on these results, we and others have proposed that comprehensive analysis of expression of all four HER family members, and their isoforms, as well as key components of their signaling networks may be necessary to improve the positive predictive value of these theragnostic and prognostic biomarker assays [3,32,36].

In this study we show that trastuzumab treatment results in the acquisition of de novo sensitivity to gefitinib or cetuximab in three of four EOC cell lines tested, implying that HER2 signaling is dispensable in these cells concomitant with compensatory EGFR signaling. While we note that HER2 expression was decreased in all three cell lines which acquired de novo drug sensitivity (T100 cells), the small number of cell lines used and single time point tested prevent us from concluding that HER2 downregulation is the mechanism of trastuzumab 'priming'. It is interesting to note, however, that complementary observations have been made in prostate cancer; gefitinib treatment of the prostate cancer cell line 22Rv1 sensitizes cells to the HER2-targeted antibody pertuzumab [37].

Our study also further highlights the differences observed between breast and ovarian cancer responsiveness to trastuzumab. Such differences are perhaps not surprising given that the progenitors of mesodermally-derived ovarian surface epithelial cells vs. ectodermally-derived breast (ductal) epithelial cells diverge early during embryonic gastrulation. It is, therefore, likely that the growth regulatory roles of HER2, as well as other HER family receptors, are divergent in these two tissues. Such functional differences may be reflected in the empirical differences observed between these tumors, such as the higher frequency of HER2 gene amplification in breast vs. EOC tumors [38]. In this context, while it is possible that long-term trastuzumab treatment results in the selection of resistant ovarian cancer subclones, we favor the hypothesis that long-term trastuzumab treatment may restrict generation-to-generation heritability of protein expression, a phenomenon recently described by Spencer et al. as a non-genetic mechanism underlying tumor heterogeneity in response to targeted therapeutics [39].

Moreover, a number of studies have demonstrated that in some HER2-positive breast carcinoma-derived cell lines, trastuzumab treatment may not directly inhibit cell growth, but still results in latent but important phenotypes. For example, the HER2-positive breast cell line JIMT-1 in vitro and in xenograft models is not significantly growth inhibited by trastuzumab [40]; however, trastuzumab does inhibit establishment of distant metastases [41]. The clinical importance of this observation is underscored by a recent study demonstrating that trastuzumab continues to improve survival even in patients who have developed apparently trastuzumab "resistant" disease [42]. In addition, other studies have demonstrated that trastuzumab sensitizes HER2-positive breast cell lines to ionizing radiation [43] and all-trans retinoic acid [44] without directly affecting cell proliferation.

In further support of this concept, our results suggest that in EOC, HER2 may potentiate but not be required for tumor cell growth, at least in a majority of cases. In the context of current terminology, this observation suggests that HER2 may not be an "addictive" oncogene in EOC [45], consistent with the prediction of Sharma and Settleman regarding 'oncogenic shock' [46,47]. The oncogenic shock hypothesis proposes that apoptosis following inhibition of an oncogene is caused by the rapid cessation of survival and growth signals with concurrent persistence of longer-lasting apoptotic signals. Our observations suggest that inhibition of a "dispensable" regulator of cell growth (in this case, HER2 in EOC) could increase reliance on another oncogene (EGFR) which, upon inhibition, could initiate oncogenic shock. In this context, one could envision a therapeutic strategy in which a tumor is "tricked" by one drug into (obligate) reliance on growth and/or survival pathways that could then be halted by a second drug. A parallel strategy has been suggested by Cao et al., wherein a signaling pathway (i.e., EGFR) is simultaneously stimulated with ligand and blocked with a specific kinase inhibitor, thereby downregulating the receptor without inducing mitogenic or survival signaling [48].

Finally, while the limited number of cell lines used in this study is insufficient to conclude that the basis for the development of de novo sensitivity to HER-targeted inhibitors is the induction of EGFR/HER3 expression by trastuzumab, here we propose that these results should be considered in the design of future ovarian cancer clinical trials. To be useful clinically, the phenomena described here must first be better understood in the patient, and particularly the kinetics of these phenomena. In the present study, the 12-week trastuzumab time course was chosen to mimic the treatment regimen of a patient who proved resistant or refractory to trastuzumab monotherapy. It may be possible to design future clinical trials to determine both the time course of changes in HER receptor expression in vivo, and/or the clinical feasibility (and kinetics) of trastuzumab "priming."

## Conclusions

In conclusion, it is possible that the disappointing results of clinical targeting of the HER axis in EOC patients stems from the intuitive, but perhaps incorrect assumption that there is a correlation between HER2 expression and responsiveness to trastuzumab. This point is supported by one recent breast cancer study which found no direct correlation between HER2 expression levels and benefit from trastuzumab therapy [33]. Similarly, there is one intriguing case report which describes remission of a patient with HER2-negative, invasive EOC following trastuzumab treatment [49]. Together, these observations suggest that ovarian cancer patients whose tumor cells express reduced, and perhaps even undetectable levels of HER2 as assessed by today's diagnostic standards, may benefit from trastuzumab "priming." Our results further indicate that SKOV-3 may not be the most representative ovarian carcinoma-derived cell line for future preclinical studies of trastuzumab in EOC, despite the historic, and nearly exclusive use of this cell line as a model for EOC in previous preclinical studies on trastuzumab [34,50-60]. In light of these new results and our improved understanding of trastuzumab's myriad effects on ovarian cancer cells, further studies to evaluate the potential clinical utility of trastuzumab in ovarian cancer patients are clearly warranted.

## Competing interests

The authors declare that they have no competing interests.

## Authors' contributions

JAW designed and conducted the studies, carried out corresponding data analyses, and drafted the manuscript. KTW participated in the studies and helped to draft the manuscript. NJM participated in study design and coordination and helped to draft the manuscript. All authors have read and approved this final manuscript.
